# Conservative treatment versus elective repair of umbilical hernia in patients with liver cirrhosis and ascites: results of a randomized controlled trial (CRUCIAL trial)

**DOI:** 10.1007/s00423-020-02033-4

**Published:** 2020-11-25

**Authors:** B. de Goede, M. M. J. van Rooijen, B. J. H. van Kempen, W. G. Polak, R. A. de Man, P. Taimr, J. F. Lange, H. J. Metselaar, G. Kazemier

**Affiliations:** 1 Department of Surgery, Erasmus University Medical Center, Room Ee-1453, PO BOX 2040, 3000 CA Rotterdam, The Netherlands; 2 Department of Surgery, IJsselland Hospital, Capelle aan den IJssel, The Netherlands; 3 Department of Epidemiology and Radiology, Erasmus University Medical Center, Rotterdam, The Netherlands; 4 Department of Gastroenterology and Hepatology, Erasmus University Medical Center, Rotterdam, The Netherlands; 5 Department of Surgery, Cancer Center Amsterdam, Amsterdam University Medical Center - VU University Amsterdam, Amsterdam, The Netherlands

**Keywords:** Umbilical hernia, Liver cirrhosis, Ascites, Liver transplantation

## Abstract

**Purpose:**

To establish optimal management of patients with an umbilical hernia complicated by liver cirrhosis and ascites.

**Methods:**

Patients with an umbilical hernia and liver cirrhosis and ascites were randomly assigned to receive either elective repair or conservative treatment. The primary endpoint was overall morbidity related to the umbilical hernia or its treatment after 24 months of follow-up. Secondary endpoints included the severity of these hernia-related complications, quality of life, and cumulative hernia recurrence rate.

**Results:**

Thirty-four patients were included in the study. Sixteen patients were randomly assigned to elective repair and 18 to conservative treatment. After 24 months, 8 patients (50%) assigned to elective repair compared to 14 patients (77.8%) assigned to conservative treatment had a complication related to the umbilical hernia or its repair. A recurrent hernia was reported in 16.7% of patients who underwent repair. For the secondary endpoint, quality of life through the physical (PCS) and mental component score (MCS) showed no significant differences between groups at 12 months of follow-up (mean difference PCS 11.95, 95% CI − 0.87 to 24.77; MCS 10.04, 95% CI − 2.78 to 22.86).

**Conclusion:**

This trial could not show a relevant difference in overall morbidity after 24 months of follow-up in favor of elective umbilical hernia repair, because of the limited number of patients included. However, elective repair of umbilical hernia in patients with liver cirrhosis and ascites appears feasible, nudging its implementation into daily practice further, particularly for patients experiencing complaints.

**Trial registration:**

Clinicaltrials.gov, NCT01421550, on 23 August 2011.

## Introduction

Umbilical hernias are common in patients with liver cirrhosis and ascites, with an incidence of up to 20% [1, 2]. The presence of ascites increases the risk of developing an umbilical hernia, due to increased abdominal pressure. Weakening of the abdominal wall, muscle wasting when nutritional status is poor, and dilatation of the umbilical vein—enlarging the pre-existent fascial opening—in patients with portal hypertension are additional contributing factors [3, 4].

Currently, there are no guidelines for the management of an umbilical hernia and its timing of surgical repair in patients with liver cirrhosis and ascites. Traditional surgical dogma dictates not to perform umbilical hernia repair under these circumstances, because of the presumed high surgical risks and high recurrence rates after surgery [5, 6]. Additionally, portal hypertension is common in cirrhotic patients with ascites, and this requires special caution because a patent umbilical vein is often present. A reopened umbilical vein can be an important outflow for the portal circulation in patients with severe portal hypertension. In these patients, elective repair without liver transplantation has been reported to result in acute portal vein thrombosis due to ligation of the umbilical vein during hernia repair, which in turn causes subsequent liver failure, necessitating emergency liver transplantation [7].

However, refraining from umbilical hernia repair under these circumstances can also result in serious complications: incarceration or evisceration of the bowel could occur, followed by necrosis of the overlying skin, necessitating emergency surgery [4, 7, 8]. Even after liver transplantation, incarceration and strangulation can still occur in untreated umbilical hernias [8]. Moreover, several studies have shown that emergency surgery is generally associated with even higher risks of morbidity and mortality compared to elective surgery, particularly in patients with liver cirrhosis [6, 8–12]. This underlines that emergency surgery in this group of frail patients should be avoided and that elective umbilical hernia repair might be the most optimal management [13, 14]. Concordingly, several retrospective and prospective series have shown good results with elective umbilical hernia repair for patients with liver cirrhosis [8, 12, 14]. However, no randomized controlled trial on this matter has been performed. The aim of this study was to compare conservative treatment with the elective repair of umbilical hernia in patients with liver cirrhosis and ascites. We hypothesized that elective repair of the umbilical hernia would result in a significant reduction of the overall complication rate and a better quality of life compared to conservative treatment.

## Methods

### Study design

The CRUCIAL trial is a randomized controlled trial conducted in two centers. Patients with liver cirrhosis and ascites older than 18 years and a primary umbilical hernia were included in the study. The presence of ascites had to be proven on imaging or had to have been drained previously. Patients were randomized into one of two groups: patients in group 1 would undergo elective surgical repair and those in group 2 would receive conservative treatment. Irrespective of the randomization group, in the case of liver transplantation, patients would receive umbilical hernia repair simultaneously if the repair had not yet been performed. Excluded from participation were patients with a recurrent umbilical hernia following a midline laparotomy in the medical history, patients who presented with American Society of Anesthesiology (ASA) score IV or above, patients with incarcerated hernia requiring emergency repair, patients with a patent umbilical vein of more than 5 mm in diameter, and patients with an expected time to liver transplantation of less than 3 months.

The study protocol was approved by the institutional medical ethical review board of the Erasmus University Medical Center, Rotterdam, before the start of inclusion. All patients gave written informed consent. An independent data and safety monitoring board was constituted before the start of the trial, consisting of two independent surgeons and one biomedical statistician. All serious adverse events were reported to the institutional review board of the Erasmus University Medical Center. The progress of the trial and adverse events were reported to the safety monitoring board.

### Randomization and masking

Patients were randomly allocated to either conservative treatment or elective repair by means of sealed, numbered envelopes that were opened in sequence. The randomization procedure was stratified for the participating center and for the model of end-stage liver disease (MELD) score ≤ 15 and > 15 and took place after collection of baseline information. Blinding for allocation did not take place for the participants, evaluators, and surgeons.

### Procedures

All repairs (elective or during liver transplantation) took place using a (separate) infra-umbilical incision, dissection (avoiding resection) of the hernia sac, and restoration of the sac and its contents into the abdominal cavity. Intra-operative resection of the sac had to be recorded on the patient’s operation report. As mesh repair has been proven to reduce recurrence rates [15], non-absorbable monofilament sutures were combined with a flat circular polypropylene mesh placed in the onlay position or in the pre-peritoneal plane. The overlap of the mesh had to be at least 3 cm in each direction. Closure of the subcutaneous tissue and skin were performed at the discretion of the surgeon.

The preferred method of anesthesia was general anesthesia, allowing for local or spinal anesthesia in the case of contra-indications for general anesthesia. Antibiotic prophylaxis was administered 10 to 30 min preoperatively.

Patients were followed up at the outpatient clinic at 2 to 3 weeks, 3 months, 12 months, and 24 months after surgery. During these visits, patients underwent physical examination, and at the 12-month visit, they underwent abdominal ultrasonography to diagnose hernia recurrence. Quality of life measurements took place at baseline, 3, 6, 12, and 24 months through the Short Form-36 (SF-36) and the EuroQoL-5D (EQ-5D) questionnaire. The pain was evaluated through the visual analogue scale (VAS), anchored by “no pain” (score of 0), and “worst imaginable pain” (score of 100) on a 100-mm scale. To avoid clustering of scores around a preferred numeric value, numbers of verbal descriptors at intermediate points were not provided.

### Outcomes

The primary outcome consisted of the hernia (and when applicable, its surgery)-related complications during 2 years of follow-up. Superficial or deep surgical site infection (SSI) [16], seroma, pneumonia, hematoma, urinary tract infection, and non-closure or delayed closure of the surgical wound at 4 weeks were considered minor complications. Major complications were mortality, evisceration, incarceration, necrosis of the overlying skin of the umbilical hernia, postoperative (> 2 weeks) leakage of ascites, liver failure, bacterial peritonitis, decompensated ascites, organ space SSI, or unexpected intensive care unit (ICU) admission related to the hernia or its repair.

These hernia-related complications were assessed for severity through the National Surgical Complication Registry (“Landelijke Heelkundige Complicatie Registratie” (LHCR)) grading tool (Table 1), scoring the maximal observed grade of hernia-related complications in each patient. Secondary endpoints were the cumulative hernia recurrence rate, pain, and quality of life.

**Table 1:** The National Surgical Complication Registry (“Landelijke Heelkundige Complicatie Registratie” (LHCR)) score

Grade	Description of complication
0	No health disadvantage, no real complication
1	Temporary health disadvantage, recovery without reoperation
2	Recovery after (re)operation
3	(Likely) permanent damage or invalidity
4	Death

### Statistical analysis

The sample size was determined at 100 patients. This calculation was based on *χ*^2^ tests with *α* = 0.05, power of 90%, and an expected decrease in overall complication rate at 2 years from 50 to 15% due to elective repair of the umbilical hernia [8]. This requires 42 patients per treatment arm, 50 when accounting for a 20% loss to follow-up.

All patients were analyzed in the group randomized to (intention-to-treat). For the primary outcome—cumulative complication rate of the umbilical hernia in the two study arms—Kaplan-Meier curves were constructed and compared with the log-rank test. The maximal observed LHCR grade was compared with the *χ*^2^ test, and secondary outcomes were analyzed through linear mixed effect models, correcting for time, randomization group, gender, time to complication, and time to liver transplantation.

Statistical analysis was performed using R statistical software (version 3.3.1). This trial is registered at Clinicaltrials.gov; number NTC01421550.

## Results

Due to unforeseen circumstances, the study was ended prematurely. Between February 2011 and July 2014, 34 patients were randomly assigned to either the intervention group (*n* = 16) or the conservative group (*n* = 18). Baseline characteristics are shown in Table 2. The median time of follow-up was 19.5 months (range 0 to 33.7 months).

**Table 2:** Baseline characteristics

	Intervention (*n* = 16)		Wait-and-see (*n* = 18)	
Gender (male, %)	11	68.8%	15	83.3%
Age^a^	57	48.8–61.5	58	56.0–60.8
BMI^a^	25.8	23.5–29.4	22.6	21.7–26.8
MELD score^b^	15.5	4.7	16.3	4.5
Hernia width^b^	2.68	2.5	1.94	1.1
Smoking	11	68.8%	6	33.3%
COPD	1	6.3%	2	11.1%
Diabetes	6	37.5%	4	22.2%
Liver failure cause				
Alcoholic hepatitis	11	68.8%	8	44.4%
PSC	1	6.3%	3	16.7%
PBC	1	6.3%	0	0%
Hepatitis B	0	0%	1	5.6%
Hepatitis C	2	12.5%	2	11.1%
Autoimmune	1	6.3%	0	0%
NASH	0	0%	2	11.1%
Other	0	0%	2	11.1%

Numbers are given in *n* (%)

^a^Median (interquartile range)

^b^Mean (standard deviation)

*BMI*, body mass index; *MELD*, model of end-stage liver disease; *COPD*, chronic obstructive pulmonary disease; *PSC*, primary sclerosing cholangitis; *PBC*, primary biliary cholangitis; *NASH*, non-alcoholic steatohepatitis

### Twenty-four-month morbidity

Eight patients (44.4%) in the conservative group received umbilical hernia repair: five received liver transplantation with simultaneous repair of the umbilical hernia, and three patients had elective or emergency repair due to complaints or incarceration. Of these eight patients, three developed recurrence of the umbilical hernia (16.7%). In the intervention group, only one patient had a recurrence of the umbilical hernia (6.3%). The mean length of stay after umbilical hernia repair was 3.21 days (range 1 to 9 days).

With regard to 24-month morbidity, 22 patients (64.7%) had at least one hernia-related complication, totaling 40 events. In the intervention group, eight patients (50%) experienced 18 events. In the wait-and-see group, 14 patients (78%) had 22 events.

**Fig 1:**
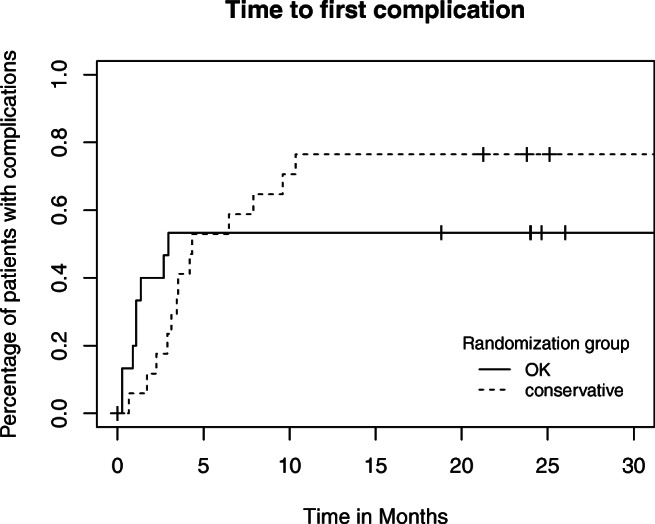
Time to first complication. This complication can be either minor or major (including death). Censored patients are marked with “+”

**Fig 2:**
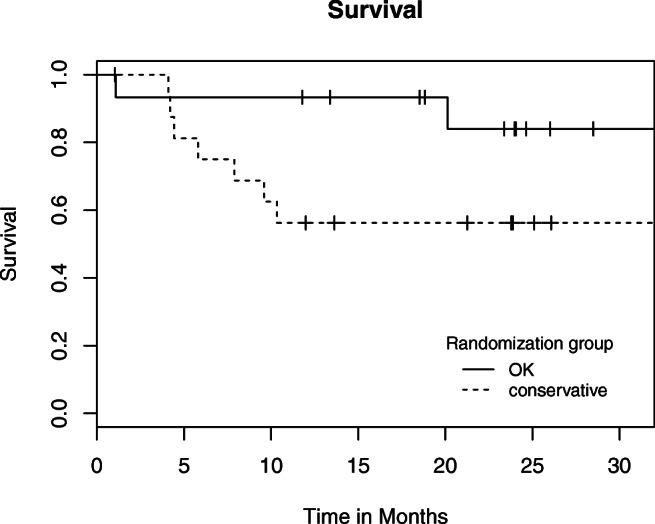
Time to death. Censored patients are marked with “+”

In Table 3, the complications are split up for minor and major complications. Figure 1 is a Kaplan-Meier depiction of the time to first event per study arm. No difference between groups was found in time to the first event (*p* = 0.663). From Table 3, the difference in mortality between groups seems rather large, yet no statistical difference was found (*p* = 0.0682). The survival is also graphically depicted in Fig. 2. In the intervention group, one death occurred due to the development of spontaneous bacterial peritonitis resulting in multi-organ failure 1 week after surgery, the other death was due to the progression of end-stage malignant disease. In the wait-and-see group, mortality was due to subcapsular bleeding after the placement of a transjugular intrahepatic portosystemic shunt in one patient; the other causes were the progression of liver disease, end-stage malignant disease, and non-surgery-related pneumonia. Additionally, no statistical significance was observed between the LHCR grades in the two groups (*p* = 0.152).

**Table 3:** Number of patients with complications, total number of complications, and maximum LHCR grade in patients with complications after 24 months

	Total (*n* = 34)	Intervention (*n* = 16)	Conservative (*n* = 18)
Patients with complication (%)	22 (64.7)	8 (50)	14 (77.8)
1 complication (%)	13 (38.2)	4 (25)	9 (50)
> 1 complication (%)	9 (26.5)	4 (25)	5 (27.8)
Total complications	40	18	22
Minor	7	3	4
SSI superficial/deep	3	1	2
Seroma	1	0	1
Hematoma	2	2	0
UTI	1	0	1
Major	33	15	18
Organ space SSI	1	1	0
Incarceration	7	3	4
Necrosis of the skin	1	1	0
Post-op. leakage	2	2	0
Bacterial peritonitis	1	1	0
Decompens. Cirr.	6	2	4
Unexp. ICU adm.	5	3	2
Death	10	2	8
Maximum LHCR grade			
1	6	4	2
2	5	2	3
3	1	0	1
4	10	2	8

*SSI*, surgical site infection; *UTI*, urinary tract infection; *Post-op.*, postoperative; *cirr*, cirrhosis; *Unexp. ICU adm*, unexpected intensive care unit admission; *LHCR*, Landelijke Heelkundige Complicatie Registratie (National Surgical Complication Registry)

When stratified for MELD score, more complications in total (*n* = 24 and *n* = 16, respectively) and more severe complications (*n* = 20 and *n* = 13, respectively) can be found in the group with patients having a score > 15, compared to the patient group with MELD score ≤ 15.

### Secondary outcomes

With regard to the quality of life measured through the SF-36, the coefficients in the linear mixed model show that over time both the physical component score (PCS) and mental component score (MCS) increase for the intervention group, compared to a much smaller increase in the PCS and even a decrease in the MCS for the conservatively treated group (as shown by the negative coefficient for the interaction between the time and randomization group in Table 4). The model further reveals that liver transplantation causes patients to score higher on both component scores. Additionally, female patients appear to have a higher MCS than their male counterparts.

**Table 4:** Coefficients from the created linear mixed models for quality of life expressed through the physical and mental component score, with their respective 95% confidence intervals

	PCS		MCS	
	Value	95% CI	Value	95% CI
(Intercept)	35.70	29.79–41.60	42.46	37.69–47.23
Time	0.34	0.09–0.59	0.32	− 0.01–0.65
Wait-and-see group	− 9.08	− 16.45 to − 1.72	− 3.63	− 9.61–2.34
Event at timepoint	0.44	− 0.74–1.61	0.06	− 1.44–1.55
LTx at timepoint	5.39	0.59–10.20	0.37	− 5.40–6.13
Female	2.27	− 5.61–10.16	8.66	3.06–14.27
Time:wait-and-see	− 0.25	− 0.66–0.15	− 0.54	− 1.05 to − 0.03

*PCS*, physical component score; *MCS*, mental component score; *CI*, confidence interval; *LTx*, liver transplantation

Quality of life measured through the EQ-5D questionnaire showed results similar to the SF-36 questionnaire. The created model showed that over time, quality of life increased (coefficient time 0.64, 95% CI 0.02–1.27). This increase was, however, smaller for the conservative group compared to the intervention group, shown by a negative coefficient (coefficient time:wait-and-see − 0.32, 95% CI − 1.33–0.69). Quality of life was at baseline already higher in the intervention group.

For pain outcomes, the coefficients in the created model show that pain decreases irrespective of the randomization group over time (coefficient time − 0.45, 95% CI − 0.99–0.09). At baseline, the scores were higher for women (coefficient 5.85, 95% CI − 12.59–24.29), and for patients in the wait-and-see group (coefficient wait-and-see 25.99, 95% CI 8.68–43.32).

Unfortunately, no cost-effectiveness analysis was performed due to the early termination of the study.

## Discussion

In this study, equal numbers of hernia-related complications were observed for elective operation and conservative approach. Additionally, elective repair and wait-and-see treatment were associated with similar quality of life and pain scores. Though underpowered, this suggests that elective repair is safe in patients with liver cirrhosis and can be performed when the patient experiences complaints from his/her umbilical hernia.

In the current study, complication rates were high in both study arms. This is in line with expectations considering the high average MELD score observed in the patient group, as a higher MELD score is associated with higher perioperative morbidity and mortality in various types of elective procedures [17–22]. In the current study, patients with a MELD score ≥ 15 experienced more incarceration, skin necrosis, and unexpected ICU admission, and had a higher mortality rate. Despite the high numbers of complications, this study shows that even in patients with a relatively high MELD score, elective repair of the umbilical hernia is safe. Additionally, a conservative approach can also be accompanied by severe complications; our study showed more incarceration, decompensated cirrhosis, and ultimately death for the patients in the conservative treatment group than in the intervention group. Other authors describe a nearly 23% emergency surgery rate due to complications of a hernia during conservative treatment [23].

The most common cause of liver failure in our study was alcoholic liver cirrhosis. Alcoholic liver cirrhosis can cause large amounts of ascites [24], which is associated with complications, including umbilical hernia [25]. This association is applicable for both groups: a large amount of ascites can cause spontaneous bacterial peritonitis in both groups [26], more frequent incarceration and wound problems in the wait-and-see group [8, 27, 28], and more recurrence and postoperative leakage of ascites in the intervention group [28–30].

However, the results from our study need to be interpreted with caution. The major limitation of this study is the small patient group, due to the premature stop of the study. As a consequence, limited data were available for modeling the secondary outcomes, resulting in large confidence intervals. Especially at later time points, many patients had dropped out of the study due to death or other causes; this was however balanced between the two randomization groups.

Another consequence of the small patient group is the chance for clustering of confounders in the randomization process. This could have caused the difference already present at baseline for quality of life and VAS scores. However, these differences could also have been due to the fact that patients were not blinded for the randomization group they were in. The fact that patients knew they would receive an operation to relieve them of their umbilical hernia might have influenced how those patients filled out their quality of life forms. Nonetheless, the effect of the baseline differences on the secondary outcomes is more than marginal and necessitates careful consideration.

Despite the premature stop of patient inclusion, this study remains a methodologically well-performed randomized controlled trial, providing a higher level of evidence than small, retrospective cohorts with their inherent bias. Therefore—unfortunately not providing a definite answer to the management of umbilical hernias in patients with cirrhosis and ascites—this study is a valuable addition to the current body of knowledge on this subject and contributes to providing transparency to the scientific community. In the context of scientific integrity, this data can prove valuable in future meta-analyses, and refraining from reporting the data adds to the burden of publication bias.

## Conclusion and implications

Despite not having enough power to show a significant difference between the two groups, this randomized controlled trial suggests that elective repair of an umbilical hernia does not cause excessive morbidity in cirrhotic patients—even with high MELD scores—and is thus advisable when the patient experiences complaints of the umbilical hernia. Considering this fact, one could argue that early elective repair when MELD scores are low—even in patients on the waiting list for transplantation—is the safer strategy. The definitive answer to whether elective umbilical hernia repair causes significantly less complications than watchful waiting in patients with liver cirrhosis remains unknown, which leaves room for further study.

## Data Availability

Not applicable
